# Service acceptance of HIV non-occupational post-exposure prophylaxis(nPEP) among college students: a cross-sectional study in China

**DOI:** 10.1186/s12889-021-11286-7

**Published:** 2021-06-24

**Authors:** Tongtong Liu, Xi Wang, Aixin Li, Jiangzhu Ye, Duo Shan, Guang Zhang, An Liu

**Affiliations:** 1 Chinese Center for Health Education, Beijing, China; 2 Beijing YouAn Hospital, Capital Medical University, Beijing, China; 3 National Center for AIDS/STD Control and Prevention, China CDC, Beijing, China

**Keywords:** HIV, AIDS, nPEP, college students, Sexual behavior

## Abstract

**Background:**

College students were the key group we should pay more attention for acquired immune deficiency syndrome (AIDS) prevention and control in recent years in China. Few studies of HIV non-occupational post-exposure prophylaxis (nPEP) knowledge and service acceptance had been conducted among them in China. This study conducted a cross-sectional survey to understand the service acceptance of nPEP and its influencing factors among college students in the three cities of China.

**Methods:**

A questionnaire survey was conducted to collect information on socio-demographic, behavioral characteristic, HIV/AIDS knowledge, nPEP knowledge, acceptance of nPEP services among the college students in Beijing, Shenzhen, and Kunming of China from March to April of 2019. Each participant completed an anonymous questionnaire on line by computer-assisted or mobile phone-assisted self-interview with informed consent. Multivariable logistic regression analyses identified predictors for service acceptance of nPEP.

**Results:**

A total of 4698 students were surveyed with the average age of 20 years old. 98.0% (4605/4698) of them were undergraduates, 21.8%(1022/4698) had sexual intercourse; 48.6% (2282/4698) heard of nPEP, among which 4.95%(113/2282) received nPEP services. The awareness rate of HIV/AIDS knowledge was 85.6% (5495/4698) with the differences statistically significant between the three cities. The awareness rate of nPEP knowledge was 16.5% (774/4698). There were significant differences in receiving nPEP services among students of different ages, genders, sexual behaviors, and knowledge of HIV/AIDS by univariate analysis. Multivariable analyses indicated that age group of 18 and under (*OR* = 2.551, 95% *CI* = 1.153–5.646), male (*OR* = 3.131, 95% *CI* = 1.866–5.253), homosexual behavior (*OR* = 4.661,95%*CI* = 2.658–8.172), heterosexual behavior (*OR* = 1.676, 95% *CI* = 1.040–2.947), no awareness of AIDS knowledge (*OR* = 3.882, 95% *CI* = 2.371–6.356) and nPEP (*OR* = 4.788, 95% *CI* = 2.50–9.169) knowledge, were associated with the service acceptance of nPEP among the college students.

**Conclusion:**

The low acceptance of nPEP services was mainly affected by low level of nPEP knowledge among the college students. Further publicity and education of nPEP knowledge were necessary, as well as promotion of knowledge of HIV/AIDS prevention and treatment. More attention should be paid to the factors associated with acceptance of nPEP services.

## Background

Acquired immune deficiency syndrome (AIDS) epidemic was at a low prevalence level in China [1]. However, the distribution of the epidemic was unbalanced, and sexual contact was the major mode of HIV transmission [2–4]. The newly reported human immunodeficiency virus (HIV) /AIDS cases among young people aged 15–24 increased from 8354 in 2008 to 16,307 in 2017, among which the proportion of students increased from 5.8% in 2008 to 18.9% in 2017, and the majority of the cases among college students were through male homosexual transmission, accounting for 57.9% [5].

According to the survey results of some parts of China, the prevalence of HIV/AIDS among college students was worrying, especially in some cities where the number of colleges and universities concentrated [6]. Since 2012, the newly reported HIV infection among college students through male homosexual transmission, has posed a challenge to HIV/AIDS prevention and control [7]. By the end of June 2017, 1244 HIV cases have been reported among students in Beijing, including 722 college students, with 98.5% of males, and 86.7% infected with HIV through male homosexual transmission [8]. Among the newly detected HIV infections and patients each year, the number of HIV infections and patients among students showed an increasing trend year by year, accounting for 1.64% of the total cases reported from January to June in 2011, up from 0.96% in 2006 [9]. Based on the data above, we concluded that parts of young students have become a key group at the risk of HIV infection in China. The main characteristics of HIV infection among young students were sexual transmission, and the majority of newly reported HIV infected was male, mainly through homosexual transmission [10, 11].

According to the previous studies on “knowledge, attitude and behavior” and behavioral characteristics, college students were sexually active, and a low rate of condom use, multiple sexual partners, casual sexual behavior, and a low awareness of sexual safety were the risk factors for HIV infection [10, 12, 13]. The time of the first sexual act among college students was getting earlier [14]. Although college students tended to be open-minded about the sexual attitudes and sexual behaviors, they lacked appropriate sexual knowledge and the ability to prevent them from sexually transmitted infections, especially HIV infection [15, 16]. In addition, with the popularity of social network software and applications, they were vulnerable to the risk factors out of the campus, prone to unsafe sex, synthetic drug abuse and other high-risk behaviors [13, 17, 18].

College students had a high level knowledge on HIV/AIDS, but poor protection awareness [19]. The result of a survey indicated that the condom use rate of the college students who had sexual experience was less than 25% [20], and students are sexually active, curious, prone to have unsafe sex and use of club drugs and other high-risk behaviors. With the increase of the proportion of sexual behaviors among college students and lack of self-protection awareness, college students were one of key groups for HIV/AIDS prevention and treatment in China [21].

Control of new HIV infection required comprehensive use of sociological, behavioral, and biomedical measures. In addition of the strategies of early detection and treatment, we needed more methods for AIDS prevention, including behavioral interventions, biomedical interventions, so as to provide more options for different needs of people. Non-occupational post-exposure prophylaxis (nPEP) referred to the provision of targeted preventive measures for the individuals at risk of being exposed to and infected with HIV through a series of services, so as to avoid their infection with HIV [22, 23].

Since early 1990s, antiretroviral treatment had prescribed for post exposure prophylaxis (PEP) following occupational exposure to HIV in many countries. This practice had since been extended to non-occupational situations, primarily for cases of sexual assault, isolated or episodic injecting drug use and consensual sexual exposure. Post-exposure prophylaxis (PEP) as a preventive treatment method helped people prevent infection or disease caused by the pathogen they were exposed to for a period of time [24].

USCDC (US Centers for Disease Control and Prevention) and WHO/ILO (World Health Organization/ International Labor Organization) respectively issued nPEP guidelines in 2005 and 2007, and recommended the use of antiviral drugs to prevent HIV infection in people at risk of sexual exposure or needle sharing. Recommended target populations for nPEP included those who had unprotected intrusive sexual behavior or needle-sharing with the source of exposure clearly HIV-infected or the state of infection unknown. The HIV antibody test of the exposed person was negative. The exposure time should not exceed 72 h [24, 25]. Healthcare workers should evaluate and provide detailed nPEP compliance counseling to explain possible adverse reactions and treatment methods emphasize the importance of follow-up, and make detailed case records and report to relevant institutions.

There was sufficient evidence to prove the effectiveness and safety of nPEP, and it had a significant effect on the reduction of new HIV infection [26]. However, the desired effect also depended on the cognition of target population on this strategy at the implementation level. HIV/AIDS prevention and control agencies in many countries have also issued authoritative guidelines, pointed out and emphasized the effectiveness of nPEP after high-risk behaviors, and recommended the use of antiretroviral drugs for nPEP to reduce new HIV infections [25, 27], which was based on previous preventive strategies such as publicity and education, condom promotion, and HIV counseling and testing. College students were the key population with concerns for HIV/AIDS prevention in recent years [20],and no relevant nPEP research had been carried out on this group in China. In this study, college students in Beijing, Kunming, and Shenzhen were recruited as the research participants to understand their behavioral characteristic, awareness of HIV/AIDS and nPEP knowledge, and acceptance of nPEP services as the potential nPEP clients.

## Methods

### Cross-sectional survey

As research target group, college students aged from 16 to 24 years old, of all grades were recruited in the universities in Beijing, Shenzhen, and Kunming. These settings were selected based on their being large cities, in different regions of China, and with more universities and colleges. College students participated in the survey voluntarily with informed consent.

In this study, the subjects were HIV antibody negative. If they were exposed to HIV because of homosexual and heterosexual behavior without condom use, instead of occupational exposure, a comprehensive evaluation would be made to determine whether to use antiretroviral drugs to prevent them from HIV infection.

This study was reviewed and approved by the Ethics Committee of the Center for STD and AIDS Prevention and Control, the Chinese Center for Disease Control and Prevention (Approval number: X190311556).

### Data collection

We employed snowball sampling to recruit participants from colleges and universities with the help of volunteers of the student societies. Initial ‘seed’ participants were recommended by volunteers, who participated in the study and then invited other students to enroll in with the informed consent. The recruitment process continued from March to April 2019.

The college students completed an anonymous questionnaire on line with the application. The questionnaire consisted of three sections, with the first of socio-demographic information, behavioral characteristics, the second of awareness of AIDS and nPEP knowledge, and the third of acceptance of nPEP services. The questionnaires were collected by five hospitals of the three cities.

With the help of college volunteers, the interviewers completed the survey at the sites of the colleges. The interviewers were healthcare workers from AIDS clinics of five hospitals. They were uniformly trained on the survey operation manual, including the questionnaire content, precautions, and interview process, to guide the interviewees to fill in the questionnaire after scanning the Quick Response (QR) code by mobile phone and instructions assigned at the scene. If the interviewees were unable to fill in by using their mobile phone, they could finish a paper questionnaire on site, and then the interviewers transferred the information to the electronic questionnaire database.

Each online questionnaire was limited to one IP address for every interviewee. After the survey was completed, all questionnaires were checked for the completeness, logical review, and correction. We also checked the questionnaires with duplicate IP addresses, to avoid those who repeated filling out the questionnaires.

### Variable definitions

AIDS knowledge of college students in this study was followed by ‘AIDS Monitoring and Evaluation Framework of China’, which included eight questions [28], 1) Can a person tell that another person is infected with HIV based on his/her appearance? 2) Can a person be infected with HIV by a mosquito bite? 3) Can a person be infected with HIV by eating with a person with HIV? 4) Can a person be infected with HIV via HIV-infected blood transfusion? 5) Can a person be infected with HIV by sharing syringes with a person with HIV? 6) Can a baby born to an HIV-positive mother be infected with HIV? 7) Can the correct condom use reduce the spread of AIDS? 8) Can a person having sex with only one partner reduce the spread of AIDS? These questions as an instrument were previously used and validated. If any six or more questions of the eight questions were answered correctly, the interviewee was defined as having awareness of HIV/AIDS knowledge.

Awareness of nPEP knowledge defined by three questions, which including 1) What do you think the role of nPEP? 2) What situations do you think require application of nPEP? 3) Do you think condom should be used during nPEP? If all the three questions are answered correctly by the interviewees, they are regarded to know basic facts of nPEP knowledge.

Acceptance of nPEP services included receiving nPEP counseling, HIV antibody testing, the prescription of a 28-day course of ART drugs, and follow-up.

### Statistical analysis

Descriptive statistics were used to describe participant’s demographic characteristics. Univariate *χ*^2^ test was used to compare the differences in the three cities, in acceptance of nPEP services among respondents with socio-demographic characteristics, HIV/AIDS prevention knowledge level, and behavioral characteristics. Only variables significant in univariate *χ*^2^analyseswere included in the multivariate logistic regression models. Multivariate logistic regression analysis was conducted to adjust odd ratios (*OR*s) for potential confounding. A stepwise forward selection procedure was used to obtain a model using a significance level for entry and exit from the model of 0.05. A *P* value < 0.05 (two-tailed) was statistically significant.

## Results

### Socio-demographic and behavioral characteristics

The number of participants from Beijing, Shenzhen, and Kunming were 2780, 826, and 1092, respectively. A total of 4698 college students with mean (*SD*) age of 20.0 (1.5) years participated in the study. Table 1 displayed the characteristics of the participants. Out of them, 71.1% (3340/4698) ranged from 19 to 21 years, 52.9% (2485/4698) were female, 98.0% (4605/4698) were undergraduates, and 79.4% (3730/4698) had no income monthly. Additionally, 21.7% (1022/4698) reported having sex experience, with 6.2% homosexual, 14.4% heterosexual, and 1.1% bisexual. A significant difference was found among socio-demographic characteristics and sexual behavior groups of the three cities (*p* < 0.001).

**Table 1:** Socio-demographic and behavioral characteristics of college students in the three cities of China, 2019

Items	Number N (%)	Cities			*χ*^2^	*P*^*#*^
		Beijing N (%)	Shenzhen N (%)	Kunming N (%)		
Age (years)	4698	2780	826	1092	720.271	< 0.001
18 and under	532 (11.3)	494 (17.8)	21 (2.5)	17 (1.5)		
19-	992 (21.1)	744 (26.8)	189 (22.9)	59 (5.4)		
20-	1343 (28.6)	671 (24.1)	276 (33.4)	396 (36.3)		
21-	1005 (21.4)	372 (13.4)	205 (24.8)	428 (39.2)		
22–24	826 (17.6)	499 (17.9)	135 (16.4)	192 (17.6)		
Gender	4698	2780	826	1092	217.211	< 0.001
Male	2213 (47.1)	1512 (54.4)	210 (25.4)	490 (44.9)		
Female	2485 (52.9)	1268 (45.6)	616 (74.6)	602 (55.1)		
Education level	4698	2780	826	1092	23.977	< 0.001
Undergraduate	4605 (98.0)	2702 (97.2)	820 (99.3)	1083 (99.2)		
Postgraduate	93 (2.0)	78 (2.8)	6 (0.7)	9 (0.8)		
Monthly income (RMB yuan)	4698	2780	826	1092	79.026	< 0.001
No income	3730 (79.4)	2184 (78.6)	595 (72.0)	951 (87.1)		
0–1000	465 (9.9)	262 (9.4)	126 (15.3)	77 (7.0)		
> 1000	503 (10.7)	334 (12.0)	105 (12.7)	64 (5.9)		
Sexual behavior	4698	2780	826	1092	62.305	< 0.001
Homosexual	293 (6.2)	133 (4.8)	47 (5.7)	113 (10.3)		
Heterosexual	676 (14.4)	446 (16.0)	105 (12.7)	125 (11.4)		
Bisexual	53 (1.1)	23 (0.9)	9 (1.1)	21 (1.9)		
No sexual*	3676 (78.3)	2178 (78.3)	665 (80.5)	833 (76.3)		

* The subject had never sexed with another man or woman

# All *p*-values are 2-sided. Comparisons with *p*-values < 0.05 were considered statistically significant

### Awareness of basic knowledge of HIV/AIDS prevention

Among college students surveyed in the three cities, 85.5% (5495/4698) answered any six or more of the eight questions correctly, and the awareness rates of college students in Beijing, Shenzhen and Kunming were 78.4, 95.0 and 96.5%, respectively. The differences among the three cities were statistically significant (*p* < 0.05). The awareness rates of the four questions, “Can a person tell that another person is infected with HIV based on his/her appearance?”, “Can a person receive HIV by a mosquito bite?”. Can a person having sex with only one partner reduce the spread of AIDS?“, and “Can a person be infected with HIV by eating with a person with HIV?” were lower than 90% (See Table 2).

**Table 2:** Knowledge of HIV/AIDS prevention among college students surveyed in the three cities of China, 2019

Questions	Number N (%)	Cities		
		Beijing (*N* = 2780)	Shenzhen (*N* = 826)	Kunming (*N* = 1092)
		N (%)	N (%)	N (%)
Can a person tell that another person be infected with HIV based on his/her appearance?				
Yes	341 (7.3)	200 (7.2)	36 (4.4)	105 (9.6)
No	3733 (79.4)	2095 (75.4)	736 (89.1)	902 (82.6)
Unknown	624 (13.3)	485 (17.4)	54 (6.5)	85 (7.8)
Can a person be infected with HIV by a mosquito bite?				
Yes	1026 (21.8)	755 (27.2)	96 (11.6)	175 (16.0)
No	3282 (69.9)	1689 (60.7)	700 (84.8)	893 (81.8)
Unknown	390 (8.3)	336 (12.1)	30 (3.6)	24 (2.2)
Can a person be infected with HIV by eating with a person with HIV?				
Yes	366 (7.8)	303 (10.9)	28 (3.4)	35 (3.2)
No	4015 (85.5)	2200 (79.1)	775 (93.8)	1040 (95.2)
Unknown	317 (6.7)	277 (10.0)	23 (2.8)	17 (1.6)
Can a person be infected with HIV via an HIV-infected blood transfusion?				
Yes	4428 (94.3)	2566 (92.3)	786 (95.2)	1076 (98.6)
No	75 (1.6)	48 (1.7)	18 (2.1)	9 (0.8)
Unknown	195 (4.1)	166 (6.0)	22 (2.7)	7 (0.6)
Can a person be infected with HIV by sharing syringes with a person with HIV?				
Yes	4473 (95.2)	2586 (93.0)	810 (98.1)	1077 (98.6)
No	51 (1.1)	42 (1.5)	5 (0.6)	4 (0.4)
Unknown	174 (3.7)	152 (5.5)	11 (1.3)	11 (1.0)
Can a baby born to an HIV-positive mother be infected with HIV?				
Yes	4284 (91.2)	2424 (87.2)	787 (95.3)	1073 (98.3)
No	82 (1.7)	66 (2.4)	10 (1.2)	6 (0.5)
Unknown	332 (7.1)	290 (10.4)	29 (3.5)	13 (1.2)
Can the correct use of condom reduce the spread of AIDS?				
Yes	4235 (90.1)	2377 (85.5)	790 (95.6)	1068 (97.8)
No	117 (2.5)	95 (3.4)	13 (1.6)	9 (0.8)
Unknown	346 (7.4)	308 (11.1)	23 (2.8)	15 (1.4)
Can a person having sex with only one partner reduce the spread of AIDS?				
Yes	3642 (77.5)	1948 (70.1)	663 (80.3)	1031 (94.4)
No	448 (9.4)	320 (11.5)	96 (11.6)	32 (2.9)
Unknown	608 (12.9)	512 (18.4)	67 (8.1)	29 (2.7)

### Awareness of nPEP knowledge

48.6% (2282/4698) of the students surveyed in the three cities heard of nPEP. The overall awareness rate of nPEP knowledge was 16.5% (774/4698), with 16.7% (463/2780) in Beijing, 11.4% (125/1092) in Kunming, 22.5% (186/826) in Shenzhen, and the proportion of students surveyed in the three cities on nPEP knowledge respectively. The difference was statistically significant (*P* < 0.001).

42.2% (963/2282) of the students who heard of nPEP thought that the use of nPEP was to prevent HIV, and 57.2% (1305/2282) considered that nPEP should be taken within 72 h after exposure. 39.3% (896/2282) thought that the price of nPEP varied according to the drug, and 97.2% (2219/2282) considered condom use during nPEP. See Table 3.

**Table 3:** Knowledge of nPEP among students surveyed in the three cities of China, 2019

Knowledge of nPEP	*N* = 2282	Cities		
		Beijing (*N* = 1310)	Shenzhen (*N* = 524)	Kunming (*N* = 448)
	N(%)	N (%)	N (%)	N (%)
What do you think is the use of nPEP?				
To treat HIV	498 (21.8)	280 (21.4)	107 (20.4)	111 (24.8)
To prevent HIV	963 (42.2)	572 (43.7)	231 (44.1)	160 (35.7)
To prevent STD	57 (2.5)	41 (3.1)	9 (1.7)	7 (1.6)
Not sure	764 (33.5)	417 (31.8)	177 (33.8)	170 (37.9)
What situations do you think require nPEP?				
Being infected with HIV	116 (5.1)	72 (5.5)	21 (4.0)	23 (5.1)
HIV-susceptible behavior occurred within the past 72 h	1305 (57.2)	777 (59.3)	299 (57.1)	229 (51.2)
HIV-susceptible behavior occurred within a month ago	36 (1.6)	23 (1.8)	8 (1.4)	5 (1.1)
Not sure	825 (36.1)	438 (33.4)	196 (37.5)	191 (42.6)
Do you think condom should be used during nPEP?				
Yes	2219 (97.2)	1260 (96.2)	518 (98.8)	441 (98.4)
No	63 (2.8)	50 (3.8)	6 (1.2)	7 (1.6)
How much does nPEP cost?				
Free	471 (20.6)	263 (20.1)	140 (26.7)	68 (15.2)
Based on the type of drug used	896 (39.3)	520 (39.7)	191 (36.5)	185 (41.3)
Not sure	915 (40.1)	527 (40.2)	193 (36.8)	195 (43.5)

### Acceptance of nPEP services and its influencing factors

9% (113/2282) received nPEP services among those heard of nPEP. There were statistically significant differences in acceptance of nPEP services among 2282 college students who heard of nPEP with different ages, gender, sexual behaviors, awareness of AIDS knowledge and nPEP knowledge (*P* < 0.001) (Table 4)

A multivariate logistic regression analysis was conducted regarding the acceptance of nPEP services among college students who heard of nPEP to determine the factors most predictive of the acceptance. The findings could provide insight into the next steps for the education and promotion of nPEP among them. After the influence of confounding variables removed, the logistic regression analysis revealed that age, gender, sexual behavior, awareness of AIDS and nPEP knowledge predicted acceptance of nPEP services among college students (*P* < 0.05). The acceptance of nPEP services of male college students was about three times(*OR* = 3.131, 95% *CIs* = 1.866–5.253) than that of females. Age was found to be a significant predictor for acceptance of nPEP services. Age group of 18 and under (*OR* = 2.551, 95% *CIs* = 1.153–5.646) had higher acceptance of nPEP services compared to the age group between 22 and 24 years old.

**Table 4:** Acceptance of nPEP services among college students with different characteristicsin the three cities of China, 2019

	Acceptance of nPEP services		
	Yes N (%)	No N (%)	*P*^*#*^
Age(years)			< 0.001
18 and under	23 (14.8)	132 (85.2)	
19-	28 (5.6)	475 (94.4)	
20-	28 (4.1)	649 (95.9)	
21-	20 (4)	481 (96)	
22–24	14 (3.1)	432 (96.9)	
Gender			< 0.001
Male	91 (8.8)	945 (91.2)	
Female	22 (1.8)	1224 (98.2)	
Level of education			0.627
Bachelor	109 (4.9)	2109 (95.1)	
Postgraduate	4 (6.3)	60 (93.8)	
Monthly income (RMB yuan)			0.181
No income	83 (4.8)	1640 (95.2)	
0–1000	9 (3.6)	243 (96.4)	
> 1000	21 (6.8)	286 (93.2)	
Sexual behavior			< 0.001
Homosexual	29 (12.2)	209 (87.8)	
Heterosexual	25 (6.8)	340 (93.2)	
Bisexual	3 (8.6)	32 (91.4)	
No sexual	56 (3.4)	1588 (96.6)	
Awareness of AIDS knowledge			< 0.001
No	39 (21.3)	144 (78.7)	
Yes	74 (3.5)	2025 (96.5)	
Awareness of nPEPknowledge			< 0.001
No	101 (6.7)	1407 (93.3)	
Yes	12 (1.6)	762 (98.4)	

^#^ All *p*-values are 2-sided. Comparisons with *p*-values < 0.05 were considered statistically significant.

Compared with those without sexual behavior, the acceptance of nPEP services was more than four times higher (*OR* = 4.661, 95% *CIs* = 2.658–8.172) among those with male homosexual behavior than those without sexual behavior, more than one time higher (*OR* = 1.676, 95% *CIs* = 1.040–2.947) among those with heterosexual behavior than those without sexual behavior.

College students without AIDS knowledge showed higher acceptance of nPEP services compared to those with the knowledge (*OR* = 3.882, 95% *CIs* = 2.371–6.356). Those without nPEP knowledge predicted higher acceptance of nPEP services compared to those with the knowledge (*OR* = 4.788, 95% *CIs* = 2.50–9.169)(Table 5).

**Table 5:** Multivariate logistic analysis of acceptance of nPEP services among college students who heard of nPEP with different characteristics in the three cities

Influence factor	B	*S.E,*	*Waldχ*^*2*^	*P*^*#*^	*OR*(95% *CI*)
Age					
22–24					1
18 and under	0.937	0.405	5.339	***0.021***	2.551 (1.153 ~ 5.646)
19-	0.657	0.362	3.298	0.069	1.928 (0.949 ~ 3.917)
20-	0.286	0.354	0.653	0.419	1.331 (0.665 ~ 2.663)
21-	0.322	0.373	0.744	0.388	1.379 (0.664 ~ 2.865)
Gender					
female					1
male	1.141	0.264	18.687	***0.000***	3.131 (1.866 ~ 5.253)
Sexual behavior					
No sexua*					1
Homosexual	1.539	0.286	28.864	***0.000***	4.661 (2.658 ~ 8.172)
Heterosexual	0.56	0.266	4.441	***0.035***	1.676 (1.040 ~ 2.947)
Bisexual	0.381	0.671	0.323	0.570	1.257 (0.393 ~ 5.452)
Awareness of AIDS knowledge					
Yes					1
No	1.356	0.252	29.075	***0.000***	3.882 (2.371 ~ 6.356)
Awareness of nPEP knowledge					
Yes					1
No	1.566	0.331	22.319	***0.000***	4.788 (2.50 ~ 9.169)

* The subject had never sexed with another man or woman

^#^ All *p*-values are 2-sided. Comparisons with *p*-values < 0.05 were considered statistically significant

## Discussion

To response HIV infections among the college students, the National Health Commission of China kept continuously strengthening the publicity and health education to reduce HIV infection risks of the students, and improved their self-protection capabilities [21, 29]. Due to the factors such as opening to sex, and being in the sexually active period, the implementation of nPEP among college students was one of the measures and strategies to prevent HIV infection after high-risk sexual behaviors [30, 31]. It is necessary for the college students to understand and be aware of the nPEP knowledge, and to receive the information of how to access nPEP services timely after their exposure to high-risk sexual behaviors.

21.7% of the surveyed students had sexual behavior and 28.7% of them were homosexual, 66.1% heterosexual, and 5.2% bisexual. The rate of sexual behavior was much less than the rate (50%) of the 15 to 24 years old who had sex with temporary partners [32], but more than the rate of self-reported sexual behavior among college students in a cross sectional study nationwide during 2010–2015, which was from 8.3 to 10% [33], and more than the result of the study in Zhejiang province of China (6.5%) [34]. The results suggest that college students who are sexually active should be regarded as one of the key groups for HIV/AIDS prevention, and receive timely and high quality sexual and reproductive health education.

This study found that the total awareness rate of AIDS knowledge among the college students in the three cities (85.6%) was higher than that of the students in Tanjin (75.8%) [35] and Harbin (72.7%) [36], which was similar to the results of the students in the survey of surveillance sentinel in Haikou (81.1–88.5%) in recent years [37]. In the three cities, the awareness rate of the college students in Beijing was significantly lower than that in the other two cities. The reason might be related to the sample structure, among which the proportion of the low age group in Beijing was higher than that of the other two cities, and the junior students had less training and health education than the senior ones. The total rate was lower than the rate required by the national health authority on the awareness rate of HIV/AIDS prevention knowledge among college students (95%) [38]. It suggests we need to further strengthen HIV/AIDS knowledge education especially for the freshmen when they were enrolling in the colleges [10].

The rates of hearing of nPEP services (48.6%), awareness of nPEP knowledge (16.5%), and acceptance of nPEP services (2.4%) among the college students, showed a trend of gradual decline. This trend showed the logical relation of KABP (knowledge, attitude, belief, practice) pattern of behavior change. The results showed the rate of the college students in this survey hearing of nPEP, which was higher than that of MSM with 24.2% in other study in Guangxi of China [39]. The rates of students knowing the role of nPEP as preventing HIV correctly and the time of initiating nPEP, within no later than 72 h after the potential exposure, were lower. These findings reflected the awareness of comprehensive knowledge of nPEP related the role and the timing of nPEP, which were the basic criteria of clinical management of nPEP [40], was weak and poor among college students. We suggest that the focus of nPEP health education and promotion in colleges should be on the introduction of key knowledge points and information of nPEP, including the role and the timing of nPEP, and how to access nPEP services.

Although the correct awareness rates of any single question about nPEP was high (42.2, 57.2, 97.2%), the level of overall rate (16.5%) was still low. This finding might be associated to health education for AIDS and nPEP knowledge in campus was not enough or not effective, and suggested that high quality and targeted nPEP knowledge publicity and education should be delivered to college students to help them to recognize the risk of HIV-related behaviors, while health education of the knowledge of HIV/AIDS prevention and control combined with publicity campaigns delivered and organized by the college administrations, student clubs or union, etc. [30, 41].

It was necessary to maintain HIV/AIDS preventive approaches such as condom use during nPEP. 97.2% of the students who heard of nPEP knew using condom while receiving nPEP. It would help college students to avoid behavioral disinhibition occurs during nPEP therapy. The behavioral disinhibition of nPEP receivers will decrease condom use or increase other unprotected sex in individuals who believed that nPEP prevented HIV transmission [42].

According to the survey, the rate of awareness of nPEP knowledge among college students who knew the knowledge of HIV/AIDS was higher than that of those who did not know. The reason may be the correlation of knowledge of AIDS and nPEP knowledge, and awareness of AIDS knowledge would promote acquisition of knowledge of HIV/AIDS. The results suggested that the content of health education in college should adapt to the new situation as the number of newly reported HIV/AIDS cases among college students increasing year by year, and the transmission route was mainly by male homosexual transmission, the prevention and control facing with more challenges in college [31].

We found the received nPEP services among college students aged 18 years and below was higher than those aged between 22 and 24 years old. The reason for this result might be that the college students aged 18 and below was usually freshmen and were just relieved from highly intensive pressure of the college entrance examination, and out of their parental supervision [13]. As they had less academic burden and more social time, students were less easily accessible to the internet and even used the dating app. by gays to find the sexual partner [43]. Therefore, it is important to develop and promote health education lessons and materials targeting to the freshmen. Therefore, it is critical to develop and promote health education lessons and materials targeting the freshman of college. We also found that the proportion of male college students receiving nPEP was higher than that of female students, suggesting that male students, especially the male freshmen had more high-risk behaviors and needed more health education and nPEP knowledge.

The proportion of college students who had sexual behavior received nPEP services were higher than no sexual students. The high proportion of receiving nPEP services indirectly reflected the high incidence of high-risk sexual behaviors among this group. It is also suggested that the group of men who have sex with men (MSM) is the main high-risk group among college students. After realizing the risk of HIV exposure or high-risk sexual behavior, they might have strong sense of seeking counseling and be willing to further accept nPEP services. It also reminded that MSM group among college students were the key people in the comprehensive intervention of AIDS prevention and control in colleges and universities, and the target population of nPEP knowledge promotion [13, 44]. With the development of economy and the frequent international cultural exchanges in recent 20 years, the Chinese had become more tolerant toward various sexual practices, sexual freedom, and openness, like homosexuality, casual sex and pre-marital sex, especially among youths [45–47]. The results of previous studies showed that high level of HIV/AIDS knowledge but more sexual partners and low rate of condom utilization existed among MSM college students [48, 49]. It is necessary and urgent to carry out effective knowledge publicity of AIDS prevention and control and nPEP knowledge in colleges, and providing the classes of health education. Because nPEP was not perceived as an alternative to safe sex, and accessing and receiving nPEP might encourage risky sexual behavior [50, 51], colleges and universities should still emphasize the importance of healthy and safe sexual behavior, especially condom use while promoting nPEP knowledge, avoiding unprotected sex in those who believed that ART prevented HIV transmission or were less concerned about transmission. MSM college students were characterized by the high-risk HIV-related sexual behaviors, such as young age of first intercourse, multi-sex partners, and low rate of condom use, group sex, commercial behavior and drug abuse [11, 52]. Particularly, they usually had high rate of AIDS knowledge, but low rate of safe sexual behavior like condom use, and more sexual partners. AIDS prevention for this group need to be paid more attentions, and effective intervening measures are urgently needed to reduce high-risk sexual behaviors.

The results of the study showed the awareness of HIV/AIDS and nPEP knowledge among the college students were factors for acceptance of nPEP services. The group without the knowledge had the higher rate of the acceptance of nPEP services than that with HIV/AIDS and nPEP knowledge, this result maybe more likely relate with their high risk sexual behavior, like not using condom or more sex partners, for without health knowledge and awareness of risk [10, 28, 53]. It is recommended that public education be promoted to increase nPEP knowledge and services for the college students, to let them know what is the nPEP, what is the time for nPEP, how to assess the risk of exposure to the infection, HIV counseling and testing, and follow-up management.

There were some limitations in this study. First, the definition of nPEP knowledge only covered three questions, including related to the potential users of nPEP, timing of nPEP and condom use during nPEP. It was not related to counseling and testing, treatment, and follow-up. The questions about nPEP knowledge need to be developed further and standardized. Second, the distribution of the college students in the study were unevenly in grades, with fewer freshmen and seniors about to graduate, and the group may have relatively high-risk sexual behavior. Third, the nPEP services should include counseling, testing and treatment. This might be because parts of the college students may only access to counseling and testing services without taking nPEP for 28 days, and they had not experienced in the whole process of nPEP. This may be because the surveys were conducted online and some participants just received counseling without taking nPEP treatment, so the number of the students receiving nPEP services may be overestimated. Forth, because the snowball sampling was used instead of common statistical sampling methods in this study, the sample was not fully representative of college students. We would further refine high-risk sexual behavior factors, such as condom use, number of sex partners, and explore the impact of behavioral factors other than sexual behavior factors on nPEP knowledge and acceptance.

## Conclusion

The college students in three cities had low rates of nPEP knowledge and acceptance of nPEP services. The acceptance of nPEP services among the group of 18 years old and below, male college student with homosexual or heterosexual behavior and with AIDS and nPEP knowledge were higher than other groups. It was suggested that the nPEP knowledge should be combined with health education and promotion of the knowledge of HIV/AIDS prevention in the college. The students may know there were nPEP as emergency measure after high-risk behaviors, how to access to the counseling and nPEP services to reduce the risk of HIV infection. Colleges and health authorities should cooperate closely to promote health education and reproductive health for HIV/AIDS prevention and control, to advocate safe sexual behavior, and to improve their self-protection awareness among college students.

## Data Availability

The datasets generated and/or analyzed during the current study are not publicly available due to ethical concerns but are available from the corresponding author on reasonable request. Available upon request to the corresponding author liuan@ccmu.edu.cn
